# High-Q microresonators on 4H-silicon-carbide-on-insulator platform for nonlinear photonics

**DOI:** 10.1038/s41377-021-00584-9

**Published:** 2021-07-05

**Authors:** Chengli Wang, Zhiwei Fang, Ailun Yi, Bingcheng Yang, Zhe Wang, Liping Zhou, Chen Shen, Yifan Zhu, Yuan Zhou, Rui Bao, Zhongxu Li, Yang Chen, Kai Huang, Jiaxiang Zhang, Ya Cheng, Xin Ou

**Affiliations:** 1 State Key Laboratory of Functional Materials for Informatics, Shanghai Institute of Microsystem and Information Technology, Chinese Academy of Sciences, 200050 Shanghai, China; 2 The Center of Materials Science and Optoelectronics Engineering, University of Chinese Academy of Sciences, 100049 Beijing, China; 3 The Extreme Optoelectromechanics Laboratory (XXL), School of Physics and Electronic Science, East China Normal University, 200241 Shanghai, China; 4 State Key Laboratory of High Field Laser Physics and CAS Center for Excellence in Ultra-intense Laser Science, Shanghai Institute of Optics and Fine Mechanics, Chinese Academy of Sciences, 201800 Shanghai, China

**Keywords:** Microresonators, High-harmonic generation, Nonlinear optics, Frequency combs

## Abstract

The realization of high-quality (Q) resonators regardless of the underpinning material platforms has been a ceaseless pursuit, because the high-Q resonators provide an extreme environment for confining light to enable observations of many nonlinear optical phenomenon with high efficiencies. Here, photonic microresonators with a mean Q factor of 6.75 × 10^6^ were demonstrated on a 4H-silicon-carbide-on-insulator (4H-SiCOI) platform, as determined by a statistical analysis of tens of resonances. Using these devices, broadband frequency conversions, including second-, third-, and fourth-harmonic generations have been observed. Cascaded Raman lasing has also been demonstrated in our SiC microresonator for the first time, to the best of our knowledge. Meanwhile, by engineering the dispersion properties of the SiC microresonator, we have achieved broadband Kerr frequency combs covering from 1300 to 1700 nm. Our demonstration represents a significant milestone in the development of SiC photonic integrated devices.

## Introduction

High-quality (Q) factor optical microresonators capable of significantly enhancing light-matter interaction have attracted strong interest in photonics community^1^. The novel photonic devices are highly in demand for both fundamental research and practical applications, such as cavity quantum electrodynamics^2^, highly sensitive sensor^3^, nonlinear devices, or filter elements^4^ for optical telecommunication systems, in which the high-Q factors are crucial for achieving high spectral resolution and sensitivity as well as strong nonlinear light-matter interaction. For highly functional optical microresonators, the important requirements of the material platforms are ultralow optical loss, wide transparent window, high index contrast, high nonlinearities, and industry compatible fabrication processes. In the past few years, we have witnessed the great success of on-chip microresonators on various photonic platforms such as Si^4^, Si_3_N_4_^5–7^, GaAs^8^, and LiNbO_3_^9–11^, etc.

Recently, silicon carbide (SiC) has generated significant attention for its superior material properties to match all the essential requirements. As a mature wide bandgap material, SiC has a wide bandgap (3.26 eV for 4H polytypes), a high refractive index (2.6 at 1550 nm) and a wide transparent window (0.37–5.6 μm)^12^, which can avoid multiple photon absorption that bothers the Si photonics. SiC is a CMOS-compatible semiconductor material which holds promise for realizing the monolithic integration of electronics and photonics with low fabrication costs via CMOS foundry^13^, giving rise to more competitiveness than LiNbO_3_ photonics. The non-centrosymmetric crystal structures of SiC grants both second-order (30 pm V^−1^) and third-order (on the order 10^−18^ m2 W^−1^) nonlinear effects^14^, and this enables an efficient light frequency conversion and on-chip generation of nonclassical light states. Moreover, unlike Si_3_N_4_ and Si, SiC exhibits the Pockels effects and thus can be used for low loss, ultrafast and wide bandwidth data transmission^15^, which is unachievable in Si_3_N_4_ and Si photonics. In addition to the above advantages, the combination with its optically-addressable spin qubits^16^, high breakdown voltage (3 × 10^−6^ V cm^−1^), high thermal conductivity (4.9 W cm^−1^ K^−1^), and high optical damage threshold (80 GW cm^−2^) further makes the SiC platform a unique and ideal candidate for realizing monolithic integration of electronics, quantum, and nonlinear photonics^17,18^.

SiC photonics has been developed for over a decade^19–25^, one of the major obstacles for the practical application is the difficulty of fabricating ultralow optical loss SiC thin films on the wafer-scale. 4H-silicon-carbide-on-insulator (4H-SiCOI) formed by ion-cutting technique has been optimized^26^. Although it has a wafer-level size, the material absorption generated by the ion-implantation-induced defects was considered as the main loss source^24^ and the Q factor is limited to below 10^5^. Up to now, it is unclear whether the SiC thin films prepared by the ion-cutting technique can be recovered to its pristine quality after the post thermal treatment. A different approach based on thin-film epitaxy techniques enabled microresonators^20,21,27^ with Qs up to 2.5 × 10^5^, which is still likely limited by the material absorption^27^. Very recently, SiC thin films prepare by thinning of bulk wafer was demonstrated to obtain Qs up to 1 million^23,25^. This method enables SiCOI substrates with the pristine material quality of bulk-SiC crystal, which represents a vital and significant progress toward the high-Q SiC photonics platform.

Here, we demonstrate an ultralow loss 4H-SiCOI platform with a record-high-Q factor of 7.1 × 10^6^. The 4H-SiCOI photonics platform was prepared by wafer-bonding and thinning techniques. The high-Q resonators were used to demonstrate various nonlinear processes including generation of multiple harmonics up to the fourth order, cascaded Raman lasing, and Kerr frequency comb. Broadband frequency conversions, including second-, third-, fourth- harmonic generation (SHG, THG, FHG) have been observed. Cascaded Raman lasing with Raman shift of 204.03 cm^−1^ has been demonstrated in SiC microresonators for the first time. Using a dispersion-engineered SiC microresonator, Kerr frequency combs covering from 1300 to 1700 nm have been achieved at a low input power of 13 mW. The Raman effect can be controlled to enable a broadband Kerr frequency combs by tuning the pump wavelength.

## Results

### High-Q SiC microresonator plaform

**Fig. 1:**
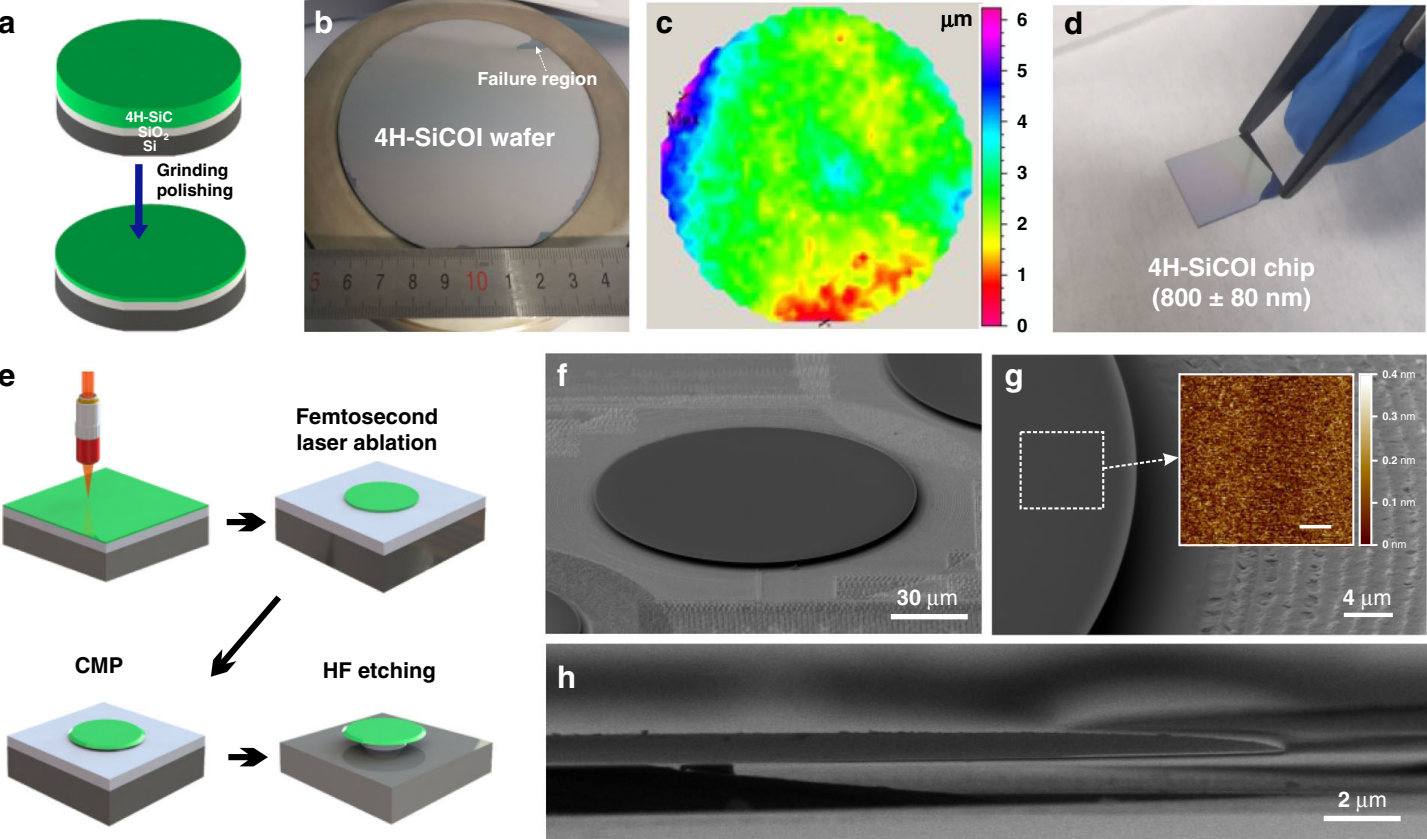
The fabrication process of 4H-SiCOI and the microresonators with highlighted features. **a** Fabrication process of pristine 4H-SiCOI material platform. **b** Photograph of a 4-inch wafer-scale 4H-SiCOI substrate fabricated using bonding and thinning method, the failure region is marked. **c** Total thickness variation of the 4H-SiCOI substrate. **d** Image of a 4H-SiCOI die. **e** Flowchart of fabricating a SiC microdisk resonator. **f** A scanning electron micrograph (SEM) of the fabricated microdisk resonator. **g** Zoom-in SEM image of the sidewall of the resonator. Inset, the atomic force micrograph (AFM) scan of the top surface of the resonator (Scale bar = 1 μm). **h** Side view SEM image of the fabricated resonator with parabolic-like shaped upper surface.

The high-Q microresonators were fabricated on a pristine 4H-SiCOI wafer. The process of the fabrication of the wafer-scale 4H-SiCOI is schematically illustrated in Fig. 1a. The process consists of bonding SiC wafer onto an oxide silicon wafer, grinding and then polishing the wafer to a thickness of several micrometers (see ‘Materials and methods’). Figure 1b shows the image of the 4H-SiCOI substrate after grinding and polishing. As marked in Fig. 1b, the bonding only fails on the edge, which is due to the weaker edge bonding strength caused by the wafer chamfering. Over 95% of SiC thin-film remained intact. As the thickness measurement shown in Fig. 1c, the fraction of uniform area within the thickness range of 2–4 µm exceeds 60%. In the next, the wafer was cut into 10 × 12 mm dies, and each die was further thinned down to the desired thickness by inductively-coupled-plasma (ICP) reactive-ion-etching (RIE) in SF_2_/O_2_ plasma and chemo-mechanical polish (CMP). The Fig. 1d shows a photograph of a 10 × 12 mm 4H-SiCOI die with SiC thickness of 800 ± 80 nm, which illustrate an improvement comparing to previous results^17,25^. The uniformity of the large area is sufficient to support an on-chip, compact photonic integrated circuit with rich functionalities^28^ in the fields of telecommunication, nonlinear optics and quantum photonics. The large thickness fluctuation during the grinding process is an industrial-level problem, which can be further reduced using foundry solutions, e.g., wafer trimming.

To investigate the optical properties of the prepared 4H-SiCOI, we fabricated microdisk resonators using a femtosecond laser-assisted chemical-mechanical polishing (CMP) method, which was developed for realizing ultrahigh-Q LiNbO_3_ resonators in previous works^29,30^. As schematically illustrated in Fig. 1e, the process begins with a 4H-SiCOI chip, followed by a femtosecond laser micromachining, CMP and hydrofluoric acid (HF) etching (see ‘Materials and methods’). Figure 1f shows a scanning electron micrograph (SEM) image of the fabricated SiC microresonator with a diameter of 160 µm. The close-up view of the edge of the resonator is shown in Fig.1g, and the root mean square (RMS) surface roughness was measured to be 0.1 nm, which reveals the achieved ultrasmooth surface and sidewall. The sidewall (upper surface) of the SiC microdisk has a parabolic-like shape as shown in Fig. 1h.

**Fig. 2:**
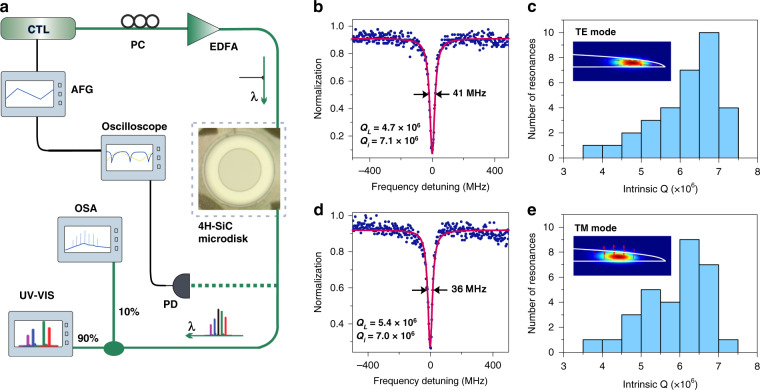
Setup and statistical study of microresonator Q factors. **a** Diagram of the measurement setup for characterizing the nonlinear optical processes in the SiC microresonator. Fundamental **b** TE and **d** TM mode resonances and their fits, with the calculated intrinsic Q values for the TE and TM modes being 7.1 × 10^6^ and 7 ×10^6^, respectively. Histogram of intrinsic Q factors for the **c** TE and **e** TM modes. The insets in **c** and **e** show the electrical field distribution from numerical simulation for the fundamental TE and TM mode. Arrows indicate electrical filed vectors. (AFG arbitray function generator, CTL continuous-wave tunable laser, PC polarization controller, EDFA erbium-doped fiber amplifier, UV-VIS ultraviolet-visible spectrometer, OSA optical spectrum analyzer, PD photodetector)

The Q factor and nonlinear optical properties of the fabricated 4H-SiC microdisk resonators were examined with the measurement setup shown in Fig. 2a. A tapered fiber with a waist of about 1 μm was used to evanescently couple the light in and out of the fabricated 4H-SiC microdisk. Light from the tunable lasers (DLC CTL 1550, TOPTICA Photonics Inc.) were sent to a polarization controller and then were amplified by an erbium-doped fiber amplifier (Beijing Keyang Optoelectronic Technology Co., Ltd.). The amplified laser was subsequently sent to the tapered fiber. A photodetector (New focus 1811, Newport Inc.), an optical spectrum analyzer (OSA: AQ6370D, YOKOGAWA Inc.) and an ultraviolet-visible spectrometer (NOVA, Shanghai Ideaoptics Corp., Ltd) were used to record the signals for characterizing the nonlinear optical properties of the fabricated 4H-SiC microdisk resonators. Figure 2b shows a typical spectral measurement at 1561.5 nm for a device having a free-spectral-range (FSR) of 2.07 nm (diameter 160 µm, thickness 800 nm). By comparing the results calculated using finite-element simulation, we identified the mode to be the fundamental transverse electric (TE) mode. The coupling conditions of the optical mode can be adjusted by tuning the coupling position finely. For example, the fundamental TE mode is nearly critically coupled is this case. The full-width-at-half-maximum (FWHM) is measured to be 41 MHz from the Lorentz fitting curve, yielding a load Q factor of 4.7 × 10^6^. By measuring the normalized transmission depth, an intrinsic Q factor of 7.1 × 10^6^ is determined. The FWHM of the transverse magnetic (TM) mode is measured to be 36 MHz, corresponding to an intrinsic Q of 7.0 × 10^6^. In a typical resonator, there are dozens of resonance peaks deriving from different mode families that have high-Q factors. To further confirm the Q measurements, histogram of intrinsic Q factors for the fundamental TE and TM mode families for several microresonators are plotted in Fig. 2c, e. The most probable value is 6.75 × 10^6^ for TE mode, while that of TM mode is 6.25 × 10^6^. To the best of our knowledge, the Q factor is the highest among the demonstrated SiC photonic microresonators so far^17,21,24,27^.

**Fig. 3:**
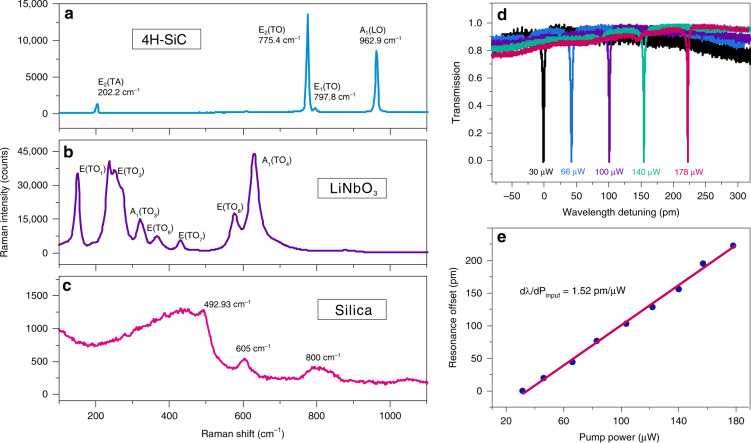
Raman and photorefractive effect in SiC. Comparison of the Raman spectra of **a** 4H-SiC, **b** LiNbO_3_, and **c** Silica. **d** Resonance continuously redshifts with increasing of the pump power. **e** Measured and fitted resonance offset of the microresonator in respect to the pump power.

Photonic platform with both χ^(2)^ and χ^(3)^ nonlinearities show greater advantages in nonlinear photonics applications, because they can be more degrees of freedom for the generation of new frequencies and provide the possibility of monolithic electro-optic modulation. LiNbO_3_ is the most famous material that simultaneously possesses large χ^(2)^ (d_33_ = 25.2 × 10^−11^ m V^−1^) and χ^(3)^ (1.6 × 10^−21^ m^2^ V^−2^) nonlinearities^9,11^. However, it is well known that LiNbO_3_ has several drawbacks such as strong Raman and photorefraction effect. SiC is a another promising material platform having both strong χ^(2)^ (d_33_ = 24 × 10^−11^ m V^−1^) and χ^(3)^ (8 × 10^−21^ m^2^ V^−2^) nonlinearities. Herein, we investigate the Raman and photorefractive effect in this SiC material. Figure 3a, b compares the Raman spectra of 4H-SiC and LiNbO_3_ measured under the same conditions. Laser power at the samples was about 1 mW for the 514.5 nm excitation. The characteristic phonon modes of 4H-SiC and LiNbO_3_ can be clearly identified in the spectra. LiNbO_3_ has several strong vibrations phonon branches with large FHWMs, and the strongest mode reaches up 44,900 counts. While in SiC, the FHWM of the dominated mode E_2_(TO) is calculated to 5.35 cm^−1^, and its intensity is about 1/3 of the strongest mode in LiNbO_3_. The lower Raman gain and narrower linewidth of Raman modes in SiC indicate that SiC is more competitive for Kerr nonlinear applications than LiNbO_3_. Figure 3c shows the Raman spectra of silica. Due to the nature of its amorphous structure, the Raman effect of silica is much weaker (about 1/10) than that of SiC. Raman effect of certain intensity in SiC is an obstacle to Kerr comb formation. It has been reported that the interfering between Raman effect and Kerr comb can be effectively suppressed by designing the FSR of the microresonator in Si and diamond^31^, and this strategy can be also used in SiC.

The photorefraction originates from the photo-induced refraction effect caused by the photoconductivity effect and the electro-optic effect, which can cause optical damage inside the crystal and hinder its application in nonlinear photonics. In optical microresonators, such as LiNbO_3_ and LiTaO_3_ microresonators, the typical feature of photorefraction is that the resonance peak blue shifts as the power increases^32^. However, in the high-Q SiC microresonator, it is found that the cavity resonance has a linear red shift relationship as show in Fig. 3d, e, with a fitted slope dλ/dP_input_ = 1.52 pm μW^−1^. These features are typical signatures of optical absorption induced thermo-refraction, implying that the thermal-optical effect is dominant in the current SiC microresonators, and the photorefractive effect is very weak. In contrast, many works have reported that there is a significant photorefractive effect in the LiNbO_3_ microresonator with a pump power of several hundred microwatts^32–34^. Therefore, we believe that SiC has more advantages than LiNbO_3_ for high optical power handling in nonlinear photonics.

### SHG, THG, and FHG generations

**Fig. 4:**
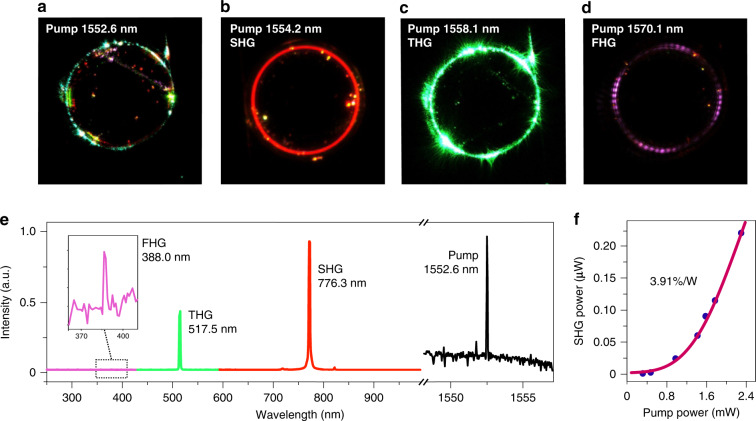
Harmonic generations in SiC microresonators. **a** Colorfully shining microresonator taken with a visible CCD camera. **b** Red, **c** green, and **d** purple harmonic light in the microresonator. The estimated pump power is 10 mW. **e** Spectra of the second-harmonic, the third-harmonic, and the fourth-harmonic signals. **f** The filled circles show the dependence of the SHG power on the fundamental power. The redline is the fitting curve for the SHG data with a normalized conversion efficiency of 3.91% W^−1^

Frequency conversions in the high-Q SiC microresonators were investigated. The multiple harmonic generations occur when the pump power is lower than that required for other nonlinear processes, such as Raman lasing and comb generation, which will be discussed in the later sections. A microresonator with a diameter of 160 μm and thickness of 800 nm was used in this section. As a proof-of-concept for harmonic generations, the microresonators were not intentionally designed for satisfying the phase matching condition. A polarization controller was used to excite the TE modes. When the pump laser wavelength was tuned between 1530 and 1570 nm, and the in-coupled power was set at 10 mW, strong emissions of various colors in the visible spectral range including red, orange, yellow, green, and purple light appear in the microdisk, which can be clearly capture by the CCD camera (see Visualization S1). Some of the shining moments of bright harmonics generation were recorded as shown in Fig. 4a, d. The bright emissions can even be spotted by naked eye (see Visualization S2). The emitted spectrum was recorded by an OSA and a spectrometer through the tapered fiber. When the pump wavelength was tuned around 1552.6 nm, a visible spectrum was recorded as shown in Fig. 4e, the emission peaks at 776.3, 517.5, and 388.0 nm can be attributed to the second, third and fourth-harmonic generation processes, respectively, since their wavelengths are exactly 1/2, 1/3, and 1/4 of the pump wavelength. Note that the ultralow photon counts of the recorded FHG is due to the low collection rate in our current experiment setup. Actually, the resonant purple light from FHG can be clearly capture by CCD camera as shown by the inset images in Fig. 4d, which reveals that the SiC microresonator exhibits the strong capability of FHG. The third and fourth-harmonic generations observed in the current work are reported for the first time in the chip-integrated SiC photonic devices.

Additionally, we also investigated the dependence of the SHG output power on the input power. The SHG power was measured by the OSA through the tapered fiber. The pump wavelength is set to be 1554.2 nm. As seen in the Fig. 4f, the SHG intensity increases with input power. A fit to the data shows that the SHG intensity is proportional to the square of the input power, as expected for the second-order nonlinear process^35^. The solid line in Fig. 4e is the fitting curve following the assumed second-order nonlinearity relationship $P_{{\mathrm{SHG}}} = \eta _{{\mathrm{SHG}}}P_{{\mathrm{Fundamental}}}^2$, where *η*_*SHG*_ is the normalized SHG conversion efficiency^35^. The fitting reveals that the *η*_*SHG*_ is about 3.91% W^−1^. It should be noted that this value is greatly limited by the fiber taper collection efficiency for the SHG wavelength. Further improvements, such as collecting the visible signal by spatial collection through objective lens^36^ or a butt/grating coupler^37^, and designing the appropriate phase matching conditions, would make it possible to increase the conversion efficiency by at least two more orders of magnitude^38^.

### Characterization of cascaded Raman Lasing

**Fig. 5:**
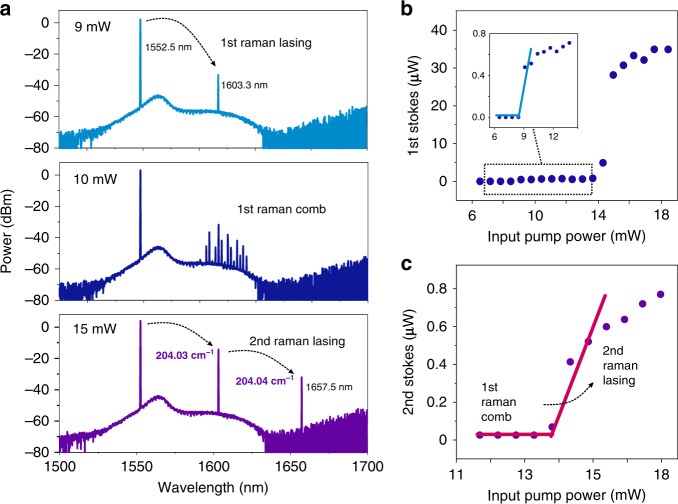
Observation of cascaded Raman lasing and threshold measurement. **a** First-order Raman lasing at 1603.3 nm with a 204.3 cm^−1^ shift from the pump (top). First-order Raman comb generation (middle). Second-order Raman lasing generation (bottom). **b** The first-order SiC Raman laser output power as a function of the input pump power. The inset is a zoom-in of the data in the dotted box. **c** The second-order SiC Raman laser output power as a function of the input pump power

For Raman lasing measurement, an amplified continuous-wave (CW) pump laser around 1552.2 nm was injected into the microresonator with diameter of 160 μm and thickness of 800 nm. The TE modes were excited by properly setting the input light polarization through the polarization controller. The power of the pump light was gradually increased, and the laser wavelength was slowly tuned to the high-Q resonance mode at the same time. As the pump power reached above 9 mW, Raman lasing at 1603.3 nm was observed in the measured optical spectrum [Fig. 5a]. The Stokes lines show the frequency shifts of 204.03 cm^−1^, corresponding to the phonon branches of E_2_(TA) in the single-crystalline SiC^39^. When the pump power was gradually increased further, the first-order Raman comb and the second-order Raman lasing appeared successively. Interestingly, these two processes cannot exist at the same time, which indicates a competition between the Raman comb and the cascaded Raman lasing. To illustrate this process, Fig. 5b, c shows the measured output first-order and second-order Raman lasing as a function of the input pump power. Figure 5b indicates that the first-order Raman lasing can be generated at two pump thresholds with the increasing pump power. The first threshold pump power is 10 mW, and the second one is 15 mW. Between the two threshold pump powers, a mini-comb is initiated around the first-order Raman lasing, and the output power of the first-order Raman lasing continues to slowly increase with increasing the pump power. As the pump power reaches about 14 mW, corresponding to the generated first-order Raman laser about 5 μW, the mini-comb disappears and the second-order Raman lasing action takes effect. The first-order laser also shows a large gain after this point, which may be due to the power transmission of the mini-comb back to first-order lasing. The output power of the second-order laser continues to increase by further enhancing the pump, while the first-order output power tends to be saturate at around 35 μW.

In the presence of Raman comb competition, the threshold pump power for the first-order Raman laser of the current SiC microresonator is measured to be 10 mW, whilst that for the cascaded second-order lasing in the same microresonator is 14 mW. Cascaded Raman lasing has been previously reported in optical fiber and other microresonators^40,41^. As a Raman-active media with the wide transparent window^12^, this is the first demonstration of simulated Raman lasing and its cascaded process in the SiC photonic structures. Combining the unique material properties of SiC, such as high thermal conductivity and high optical damage threshold, the realization of the cascaded SiC Raman laser will offer a new opportunity to extend the spectral coverage of traditional laser light sources.

### Kerr comb generation

**Fig. 6:**
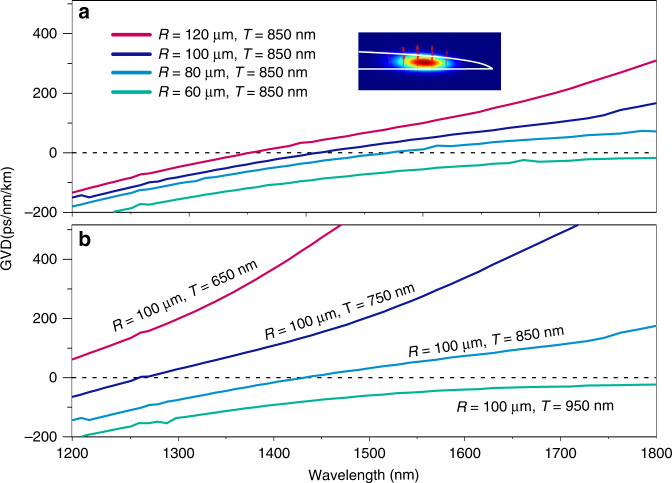
Dispersion engineering of the microresonators. **a** Dispersion calculation for the fundamental TM mode of the microresonators with a thickness of 850 nm and radius of 60, 80, 100, and 120 μm. Inset show the simulated mode profile of the fundamental TM mode. **b** Dispersion calculation for the fundamental TM mode of the microresonators with a radius of 100 μm and thickness of 650, 750, 850, and 950 μm

In order to generate the broadband frequency comb, the record-high-Q factor of the current SiC microresonator facilitates generation of the optical parametric oscillation (OPO) followed by the cascaded frequency conversion processes of high efficiency thank to the low intracavity losses^42^. Besides, an anomalous group-velocity dispersion of the microresonator is also required to compensate for the nonlinear phase shift induce by self-phase modulation and cross-phase modulation^43^. To investigate the dispersion properties of the fabricated microresonator, we theoretically calculated the group-velocity dispersion (GVD) using a finite-element mode solver. The dispersion calculations include material anisotropy for TE and TM mode. Figure 6a shows the dispersion for the fundamental TM mode of the microresonators with different radius of 60, 80, 100, and 120 μm, obviously, the dispersion curves increase with the increasing the resonator radius and can be tuned from normal dispersion to anomalous. Next, the thicknesses of 650, 750, 850, and 950 nm were compared at the fixed radius of 100 μm as shown in Fig. 6b. The calculation results show that the thinner microdisks provide greater anomalous dispersions. The SiC microresonators show the variable dispersion for the fundamental TM mode from normal to anomalous dispersion by controlling its thickness and radius. Note that the fundamental TE mode of the microresonator cannot be engineered to reach the anomalous dispersion regime. Therefore, only the TM mode are excited in the following experiments for the Kerr comb generation. A microresonator with a radius of 100 μm and a thickness of 850 nm is chosen to meet the anomalous dispersion according to Fig. 6.

**Fig. 7:**
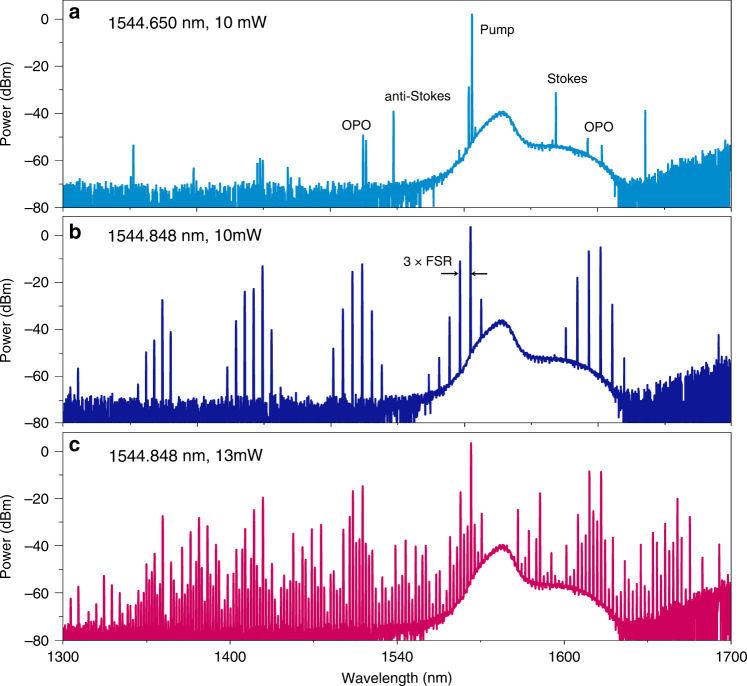
Broadband Kerr frequency comb generations in a 4H-SiC microresonator with a radius of 100 um and thickness of 850 nm. **a** Measured OPO spectra generated with a launched pump power of 10 mW. **b** Hyper-OPO spectra generation when red-tuned the pump wavelength into resonance near 1544.848 nm. **c** Broadband Kerr frequency comb generations when a 13 mW pump was injected into the microresonator at 1544.848 nm

The Kerr frequency comb is generated through the OPO process, which depends on the combination of parametric amplification and oscillation of nonlinear four wave mixing in microresonators. If a CW is injected and tuned into a high-Q resonance, strong light field will be accumulated inside the microresonator. As the parametric gain exceeds the loss in a round-trip of the cavity, the accumulated power triggers OPO at the critical power threshold^44^. The OPO threshold power can be estimated by the expression^45^ $P_{{\mathrm{th}}} \approx 1.54\left( {\frac{\pi }{2}} \right)\frac{{Q_c}}{{2Q_L^3}}\frac{{n^2LA_{eff}}}{{\lambda n_2}}$, where *A*_*eff*_ ≈ 2.5 μm^2^ is the effective area of the microresonator, *n* = 2.6 and *n*_2_ = 8 × 10^−19^ m^2^W^−1^ are the refractive index and the Kerr nonlinearity coefficient of SiC. For a microresonator with a radius of 100 μm and a load Q factor of 4 × 10^6^, the calculated threshold power is 5.2 mW when near critical coupling condition. Figure 7a shows the measured spectra of the initial OPO state, when the pump wavelength at 1544.65 nm. The first-order Stokes, anti-Stokes and second-order Raman occur, which can be confirmed according to the corresponded wavelength shift of 204.03 cm^−1^. The OPO threshold power is measured to be around 10 mW, which is two times the theoretical value. These may because the existence of the Raman scattering causes the pump power to be partially used to excite the OPO process. When the pump wavelength is gradually red-tuned into resonance near 1544.848 nm, the Raman-related signal vanishes but OPO oscillation persists, which indicate a power transfer between OPO and Raman oscillations. This power exchange is controllable and reversible by adjusting the pump frequency^46^. The OPO undergoes higher power gain until self-stabilized by thermal locking, which triggers more comb lines with a spectral spacing three times wider than the FSR. By further enhancing the pump power, the gaps between the primary sideband can be fulfilled by comb lines with spacing of one FSR (2.08 nm around 1550 nm). A broadband Kerr frequency comb spanning from 1300 to 1700 nm was measured as illustrated in Fig. 7c. The shape of the observed combs indicates that the generated combs are modulation instability (MI) frequency combs^47^. For actual application, the next essential step is to access soliton formation. The thermo-optic coefficient of SiC (4.21 × 10^−5^ K^−1^) is on the same order of magnitude compared with that of Si_3_N_4_ (2.4 × 10^−5^ K^−1^), and the Raman effects have been avoided in the current MI combs. The combination of the facts provides great prospect for soliton generation in SiC platform by using temporal scanning techniques, which has been widely demonstrated in other material platforms^47,48^.

## Discussion

In conclusion, we have reported a low loss, dispersion-engineered SiC microresonator, with the potential scalability to wafer-scale. A mean Q factor 6.75 × 10^6^ was determined by a statistical analysis of tens of resonances. Using these devices with a Q factor up to 7.1 × 10^6^, we have demonstrated on-chip SHG with a conversion efficiency of 3.91% W^−1^ even without optimization of the phase matching. The third and fourth-harmonic generations were observed for the first time in on-chip SiC photonics devices, which can be determined by the recorded spectrum and the bright color light emitted from the resonators. Cascaded Raman lasing has also demonstrated in SiC microresonators for the first time. The threshold pump powers were measured to be 10 and 14 mW for the first-order and the cascaded second-order Raman lasing, respectively. Finally, low-threshold OPO and broadband (~400 nm) Kerr frequency combs were achieved using a dispersion-engineered SiC microresonator. We believe that the high-Q SiC photonics platform and its diverse nonlinear functionalities will pave the way to a wide range of quantum and classic applications based on 4H-SiCOI.

## Materials and methods

### 4H-SiCOI fabrication

The process of the fabrication of the wafer-scale 4H-SiCOI is schematically illustrated in Fig. 1a. The 4-inch high-purity semi-insulating 4H-SiC wafer and the thermally oxidized Si (100) substrate were directly bonded at room temperature to form bulk-SiC-SiO_2_-Si structure. Plasma surface activation was used in this process. In order to enhance the bonding strength, the bonded wafer was annealed at 600 °C in N_2_ atmosphere for 8 h. Then, the bonded wafer was processed by mechanical grinding to thin the SiC layer from a thickness of 500 μm to sub 10 μm. The grinding process involves using a diamond-resin bonded wheel to mechanical remove the SiC layer. Finally, the wafer was cut into 10 × 12 mm chips, and each chip was further thinned to the predesignated thickness by inductively-coupled-plasma (ICP) reactive-ion-etching (RIE) in SF_2_/O_2_ plasma and chemo-mechanical polish (CMP).

### Microresonator fabrication

As schematically illustrated in Fig. 1e, the prepared 4H-SiCOI structure is a layer of SiC (800 nm) on top of a buried silicon oxide (2 μm) layer on silicon substrate. To pattern the resonator, femtosecond laser micromachining was employed. This method has some unique characteristics including nonthermal ablation, high spatial resolution combined with decent material removal rates, as well as flexibility in generating arbitrary patterns in the mask-less direct write fashion. The femtosecond laser beam was focused into a ∼1 μm diameter focal spot using an objective lens (×100/NA 0.7), and the micromachining was carried out at a scan speed of 10 mm s^−1^ of the focused laser spot. It is noteworthy that femtosecond laser ablation generally leaves behind a surface roughness on the order of ∼100 nm, which should be eliminated for fabricating high-Q microresonators. The CMP process was performed to smooth the top surface and sidewall of the 4H-SiC microdisk using a wafer lapping polishing machine. The CMP process allows to achieve an extremely low surface roughness of 0.1 nm at the edge of 4H-SiC microdisk, which is vital for achieving ultrahigh-Q factors. Lastly, the suspended microdisk was formed by undercutting the silica layer into the pedestal in a diluted HF solution (10%).

### Q factor, harmonics, Raman lasing, and Comb characterization

A C-band continuous-wave tunable laser (DLC CTL 1550, TOPTICA Photonics Inc.) was used as both the signal (for measuring Q) and pump (for exciting various nonlinear processes) source. The fine tuning of the laser was controlled by an arbitrary function generator (AFG3052C Tektronix Inc.). The polarization state of the tunable laser was adjusted by a fiber polarization controller (FPC562, Thorlabs Inc.). The tunable laser was amplified by an erbium-doped fiber amplifier (KY-EDFA-HP-37-D-FA, Beijing Keyang Optoelectronic Technology Co., Ltd.). A tapered fiber with a waist of 1 μm was used to evanescently couple the light into and out of the fabricated 4H-SiC microdisk. A photodetector (New focus 1811, Newport Inc.) was used to record the signal from the tapered fiber and to convert the optical signal to electrical signal. The electrical signal was further sent to an oscilloscope (MDO3104 Tektronix Inc.) for the Q factor measurement of the 4H-SiC microdisk resonator. To characterize the nonlinear optical properties of the fabricated 4H-SiC microdisk resonators, 90% of the output beam from the coupling fiber was directed to an optical spectrum analyzer (OSA: AQ6370D, YOKOGAWA Inc.) for infrared spectral analysis using a fiber beam splitter, whereas the remaining 10% of the output beam was routed to an ultraviolet-visible spectrometer (NOVA, Shanghai Ideaoptics Corp., Ltd) for ultraviolet-visible spectral analysis.

## Supplementary information

Visualization S1

Visualization S2
